# Child Weight Status: The Role of Feeding Styles and Highly Motivated Eating in Children

**DOI:** 10.3390/children10030507

**Published:** 2023-03-04

**Authors:** Maria A. Papaioannou, Thomas G. Power, Teresia M. O’Connor, Jennifer O. Fisher, Nilda E. Micheli, Sheryl O. Hughes

**Affiliations:** 1 Department of Pediatrics, USDA/ARS Children’s Nutrition Research Center, Baylor College of Medicine, Houston, TX 77030, USA; 2 Department of Human Development, Washington State University, Pullman, WA 99164, USA; 3 Department of Social and Behavioral Sciences, Center for Obesity Research and Education, Temple University, Philadelphia, PA 19122, USA

**Keywords:** Hispanic families, feeding styles, child eating behaviors, child weight status, eating in the absence of hunger, food motivation

## Abstract

Although parental feeding plays an important role in child eating and weight status, high food motivation among children may also be a factor shaping how feeding impacts child weight. This study explored whether individual differences in preschool children’s food motivation interacted with mothers’ feeding styles in predicting subsequent child weight status. Participants included 129 Hispanic Head Start mother/child dyads. Data were collected at ages 4–5 years (Time 1) and 7–9 (Time 3). Staff measured heights/weights and observed children in an eating in the absence of hunger task. Mothers reported on feeding styles/practices and children’s eating behaviors. A principal components analysis derived a measure of highly motivated eating in children. Multiple regressions predicted Time 3 child BMI *z*-scores. Time 3 BMI *z*-scores were positively predicted by authoritative and indulgent feeding styles and negatively predicted by monitoring. Since feeding style interacted with highly motivated eating, separate regressions were run for high and low food motivation in children. Unexpectedly, results showed that authoritative feeding positively predicted Time 3 child BMI *z*-scores only for children showing *low levels* of food motivation. Characterizing differential parental feeding and child eating phenotypes may assist in tailoring childhood obesity prevention programs for the target populations.

## 1. Introduction

Overweight and obesity remain high among children in the U.S. [1,2], with disproportionately higher rates seen among ethnically diverse populations [2,3]. Disparities and subsequent health burdens among diverse ethnic groups are a major health concern in the U.S. Roughly 30% of Hispanic children have overweight or obesity by preschool age, with the prevalence increasing to 46% at ages six to eleven years [3]. In order to appropriately inform obesity prevention programs for Hispanic children, evidence is needed that represents the cultural experience of the parent/child feeding experience among these racial/ethnic minority families.

Parents play a key role in the development of child eating behaviors and subsequent weight status through the various feeding styles [4] and practices [5] used to socialize children during eating episodes. Whereas feeding styles capture the global approach and emotional climate in which feeding takes place [6,7], feeding practices reflect specific, goal-directed behaviors used to direct child eating [5,8]. Feeding styles are believed to be enduring and trait-like, whereas feeding practices are thought to be modifiable [8]. Numerous studies (Hughes and Power [4] provide a review) demonstrate associations between an indulgent feeding style (i.e., low control paired with high responsiveness) and the highest overweight and/or obesity risk among children in Hispanic families with low-incomes. Moreover, general parenting [9] and feeding styles (i.e., a global approach) [7] characterized by high levels of control and low responsiveness (i.e., authoritarian styles) in this population may have protective influences on child weight status. Benefits of high control in feeding among racial/ethnic minority families are consistent with Domenech et al.’s [10] ‘protective parenting’ concept (i.e., low autonomy granting and high demandingness). These observations suggest that high levels compared to low levels of parental control may be optimal for obesity prevention among some children who may be more responsive to food and its characteristics among racial and ethnic minority families. 

High food motivation is thought to be a dimension of appetite regulation in children [11,12] that shapes hunger and satiety responses and the quantity/composition of consumption. A wide range of food motivated behaviors have been associated with higher weight status among young children [13,14], including parent report of children’s enjoyment of food and food responsiveness [15,16,17,18,19,20] as well as direct observations of eating in the absence of hunger [21,22,23,24]. Importantly, food motivated behaviors have a strong genetic component [25,26] and show stability over time [23,27]. Multiple studies support the premise that appetitive phenotypes among children confer behavioral susceptibility to obesity [20,28], suggesting that children with higher food motivation exhibit greater susceptibility to obesogenic influences, including snacking [29], consumption of larger portion sizes [30], intake of highly processed foods [31], as well as greater energy intake, in general [23,32,33].

Developmental psychologists have long recognized that different children benefit from different types of parenting [34,35]. However, the feeding literature has been remarkably one-sided in its perspective, focusing only on the effects of what parents do. The need for a more nuanced understanding of child contributions to parental feeding has been recently acknowledged with a call for more research focused on ‘precision approaches to feeding children’ [36]. To date, evidence on the influence of parental feeding on specific child eating behaviors is limited, with only one study showing that children with a higher food approach had higher BMI *z*-scores when considering parental feeding behaviors [37]. Beyond this one study, little consideration has been given to individual differences in food motivation among children that may increase their obesity risk [13,38].

The aim of the current study was to examine, in a study of Hispanic families with low-income levels, the degree to which individual differences in children’s food motivation in the preschool years interacted with mothers’ feeding styles in predicting subsequent weight status in children. The current paper involves further analyses of data from a longitudinal study showing that indulgent and authoritative feeding in the preschool years was associated with higher child weight status in elementary school [39]. In these analyses, we used both maternal reports [40] and observations of child behavior [41] to assess highly food motivated eating. We hypothesized that parental feeding style would be a stronger predictor of later child weight status for children showing highly motivated eating patterns. Specifically, we expected that low levels of parental control (i.e., indulgent feeding styles) in conjunction with highly motivated eating in children would predict higher child weight status in the elementary school years.

## 2. Materials and Methods

### 2.1. Participants

A total of 129 Hispanic parents and their 4–5-year-old children residing in a large urban city in the southern part of the U.S. were included in this study. These families participated in a larger longitudinal study (*n* = 187) that examined eating behaviors of children from families with low incomes [39]. Parent/child dyads were eligible to participate if the parent self-identified as Hispanic and spoke either English or Spanish, and the child was attending Head Start. Parent/child dyads were not eligible to participate if either the parent or child had dietary restrictions for any reason, such as diabetes, food allergies, or were following a special diet. Additionally, children that had developmental problems, such as autism or other significant developmental delays, were excluded as it would have limited their ability to perform the study tasks. This study was reviewed and approved by the Institutional Review Board at Baylor College of Medicine (ethics approval number H-26796). Before any study activities took place, study staff explained the purpose of the study to parents in either English or Spanish (their language of choice). Consent was obtained for parents’ participation and verbal assent was obtained for children’s participation. All consenting parents were mothers; therefore, parents will be referred to as ‘mothers’ hereafter.

The larger longitudinal study included 187 mother/child dyads (i.e., original sample) [42]. Eighteen months following baseline assessments (*M* = 18.39, *SD* = 1.58), follow-up assessments (Time 2) were conducted on 144 mother/child dyads. Approximately 24 months after Time 2 (*M* = 23.6, *SD* = 6.54), Time 3 assessments were conducted on 129 mother/child dyads. Only data from baseline (Time 1) and the Time 3 are included in the present study. Data on all the variables, that were needed for analyses, were available for 129 mother/child dyads. The baseline demographics of the 129 mothers are presented in Table 1. The mean age of the mothers was 31.55 years (*SD* = 6.6). The majority of the mothers were unemployed (79.1%), born in Mexico (63.5%) or Central America (17.9%), and married (58.9%). The educational status of the mothers ranged from 6th grade to beyond college. The ages of the children at Times 1 and 3 assessments were *M* = 4.76 (*SD* = 0.46) and *M* = 8.34 (*SD* = 0.71), respectively. Approximately half of the children were male (46.5%) and had a healthy weight status (48.8%). About 22.5% of the children were classified in the overweight category and 27.1% were classified in the obese category. The percentage of children with overweight and obesity in this study is higher than that of 2- to 5-year-old Hispanic children with overweight or obesity in the U.S. (i.e., 30%) [3]. There were no significant differences regarding demographic variables between the initial sample of 187 mother/child dyads and the 129 mother/child dyads with available data at both time points (Times 1 and 3; Table 1). Participants in this study may be representative of Hispanics in this geographical area.

### 2.2. Measures

All questionnaires used in this study were translated into Spanish and back translated into English to assure understanding of the wording and concepts. These questionnaires have been used successfully in previous studies with Hispanic participants. All measures were completed at Time 1 (baseline) except for the child anthropometrics, which were completed at both Time 1 (baseline) and Time 3 (approximately 42 months after baseline).

#### 2.2.1. Caregiver’s Feeding Styles Questionnaire (CFSQ)

The CFSQ is a well-established 19-item questionnaire developed by Hughes and colleagues [7] to measure feeding styles for use with Hispanic parents of young children from families with low-income levels. The CFSQ uses a 5-point Likert scale, ranging from never to always. A cross-classification of scores on dimensions of demandingness and responsiveness identifies four feeding style categories as follows: authoritarian (high demand/low response); authoritative (high demand/high response); indulgent (low demand/high response); and uninvolved (low demand/low response). Evidence of test-retest reliability, internal consistency, convergent, and predictive validity has been obtained with ethnically diverse families, including Hispanic, with low incomes [4,43,44,45,46,47,48,49,50,51,52,53,54,55,56,57,58,59].

#### 2.2.2. Child Feeding Questionnaire (CFQ)

The CFQ is a validated questionnaire used to assess feeding attitudes and practices [60]. The CFQ measures four attitudes (perceived responsibility, perceived child weight, perceived parent weight, and concern about child weight) and three practices (restriction, pressure to eat, and monitoring). In the current study, only the following subscales were used as they assess feeding practices: restriction (e.g., I intentionally keep some foods out of my child’s reach); pressure to eat (e.g., my child should always eat all the food on her plate); and monitoring (e.g., how much do you keep track of the high fat foods that your child eats?). This questionnaire has been used and validated in low-income samples [7,54,61,62,63].

#### 2.2.3. Children’s Eating Behavior Questionnaire (CEBQ)

The CEBQ measures child eating behaviors. It contains 35-items with a 5-point Likert-type response scale, ranging from never to always [40]. Four subscales assess food approach behaviors (Food Responsiveness, Enjoyment of Food, Desire to Drink, Emotional Overeating) and four assess food avoidant behaviors (Satiety Responsiveness, Emotional Undereating, Slowness in Eating, Food Fussiness). Multiple studies with cross- sectional and longitudinal designs support the predictive validity of the measure through robust associations of CEBQ subscales with weight status among young children [18,64,65,66,67,68,69,70,71,72]. The CEBQ scores were used in this study, in part, to measure the highly motivated eating construct in children. 

#### 2.2.4. Eating in the Absence of Hunger Task (EAH)

This task was developed by Fisher and Birch [41] to measure child eating beyond satiation. Higher scores have been associated with higher child weight status across multiple studies ([24,73,74,75,76], also see Lansigan et al. [14] for a review). In order to minimize hunger prior to the task, children were provided with a standardized meal of palatable foods accounting for 40% of the estimated daily food energy needs of a four- to five-year-old. After the meal, children were interviewed individually to determine fullness. Each child was then left alone with age-appropriate toys and sweet and savory snacks (i.e., potato chips, Skittles, pretzels, sherbet, ice-cream, Hershey bars, and chocolate chip cookies) for ten minutes while being observed remotely. Scores for each child on this task reflected the total number of kilocalories eaten in the absence of hunger based on weighed food intake. Final scores across the children were highly positively skewed. Thus, data were recoded into three values: 1 = less than 20 kilocalories (*n* = 37); 2 = 20 to 125 kilocalories (*n* = 74); 3 = greater than 125 kilocalories (*n* = 75). High values reflected higher levels of eating in the absence of hunger. The first group was defined as children who ate no food or ate a very minimal amount (the distribution had a natural break at 20 kilocalories); the second and third groups were defined by a median split of the remaining children. EAH scores were used in this study to measure the highly motivated eating construct in children.

#### 2.2.5. Anthropometrics

Trained research staff took child height and weight measurements following a standard protocol [77]. Children were weighed in duplicate using a digital weight scale (Health-O-Meter model 752 KL, Health O Meter, China) to the nearest 0.1 kg, and height was measured in duplicate using a stadiometer (Seca model 214, Seca, China) to the nearest 0.1 cm. Using the Centers for Disease Control and Prevention Reference Standards, age- and gender-specific Body Mass Index (BMI) standardized scores (BMI *z*-score) were calculated [78]. The following weight status categories were used for the children: underweight (BMI < 5th percentile), healthy weight (BMI ≥ 5th to < 85th percentile), overweight (BMI ≥ 85th to < 95th percentile), or obese (BMI ≥ 95th percentile).

### 2.3. Data Analyses

All analyses were run using the Statistical Package for the Social Sciences (SPSS, Version 28.0, Chicago, IL, USA). First, we conducted a principal components analysis on the eight CEBQ subscale scores and the child EAH score to derive a measure of highly motivated eating in children. To maximize the sample size, all Time 1 data were used in this analysis (*n*’s = 187 for the CEBQ and 186 for EAH). Mean scores were calculated on CEBQ subscales if mothers completed all items. If mothers completed at least 75% of the items on a given subscale, then the score for that subscale was calculated by examining the mean of the non-missing items. If a mother completed less than 75% of the items, then the score was considered missing for that subscale. The main analyses were conducted through multiple regression. For the longitudinal analysis, to allow for comparison with Hughes et al. [39], we only analyzed data from the mothers (*n* = 129) and children (*n* = 128) who completed all relevant assessments at Times 1 and 3. Multiple regressions were conducted to predict the child BMI *z*-score at Time 3 from those Time 1 measures, showing significant prediction in the previous analysis of this dataset reported in Hughes et al. [39]. Based on these previous analyses, predictors were: (1) child BMI *z*-score; (2) CFQ feeding practices (three scores—restriction, pressure to eat, and monitoring); and (3) CFSQ feeding styles (one dichotomous predictor for each of three feeding styles—authoritarian, authoritative, and indulgent; uninvolved feeding served as the reference group). Additional predictors for these regressions were the highly motivated eating score on the children and the three feeding styles by highly motivated eating interactions. As described below, because of the results of the preliminary analyses, separate regressions were run for highly motivated eating in children derived from the CEBQ and the EAH task.

## 3. Results

The principal components analysis on the combined CEBQ and EAH measures for highly motivated eating in children yielded three components—a first component with loadings on six of the eight CEBQ subscales that assessed highly motivated eating, a second component that primarily assessed emotional eating, and a third component assessing EAH. EAH scores loaded a separate component in this analysis and only showed a significant correlation with one of the eight CEBQ subscales, emotional overeating, r(184) = 0.15, *p* < 0.05. Given that the EAH was not highly correlated with the CEBQ scores, we ran separate regressions for the CEBQ-based and the EAH-based assessments of highly motivated eating in children. To derive the CEBQ measure, we reran the principal components analysis specifying only one component. This component accounted for 30.95% of the variance in CEBQ subscale scores. The loadings are presented in Table 2. The highly motivated eating score was calculated by taking the mean of the five CEBQ subscales with loadings > 0.30 (reverse scoring those subscales with negative loadings). Coefficient alpha for this five-item scale was 0.69.

Table 3 presents the descriptive statistics and correlations for all variables used in the regression analyses. The Time 1 CEBQ measure of highly motivated eating in children was positively correlated with Time 1 indulgent feeding and Time 1 child BMI *z*-score and negatively correlated with Time 1 authoritarian feeding. EAH was positively correlated with child BMI *z*-scores at both Time 1 and Time 3 and not significantly correlated with feeding styles or practices. Finally, Time 1 indulgent feeding style was positively correlated with child BMI *z*-scores at both time points and Time 1 authoritarian feeding negatively correlated with child BMI *z*-scores at Time 1.

Table 4 presents the results of the multiple regression predicting child BMI *z*-scores at Time 3 using the CEBQ measure of highly motivated eating in children. For the entire sample, controlling for Time 1 BMI *z*-score, Time 3 BMI *z*-scores was positively predicted by authoritative and indulgent feeding styles and negatively predicted by monitoring. As was the case for the bivariate correlations, highly motivated eating at Time 1 did not significantly predict Time 3 child BMI *z*-scores. However, the authoritative feeding by highly motivated eating interaction was significant. Because authoritative feeding style significantly interacted with highly motivated eating, separate regressions were run on children above and below the median on highly motivated eating. As shown in the two right hand columns of Table 4, in addition to the Time 1 child BMI *z*-scores, the predictors identified in the sample as a whole were only significant for children below the median on highly motivated eating.

The regression replacing the CEBQ highly motivated eating score with the EAH score yielded the same significant predictors as the previous analysis (predicting Time 3 child BMI *z*-scores from CEBQ highly motivated eating). The regression predicting the Time 3 child BMI *z*-scores from the EAH motivated eating score showed feeding practices (i.e., monitoring) and feeding style (authoritative and indulgent) as significant predictors. There was no significant main effect for EAH nor any significant EAH by feeding style interactions.

## 4. Discussion

The present study further analyzed data from a longitudinal study among Hispanic families that demonstrated associations between authoritative and indulgent feeding styles in mothers of preschoolers and later higher weight status in elementary school-aged children [39]. Specifically, the purpose of this study was to investigate the extent to which children’s individual differences in food motivation, as measured by maternal report and observations, interacted with maternal feeding styles in the prediction of children’s later weight status. Unexpectedly, later child weight status was positively predicted by authoritative feeding (characterized by high levels of parental control and responsiveness) but only for children *below* the median on highly motivated eating as reported by mothers.

Developmental psychologists have posited, and the feeding literature has shown, that children can benefit from tailoring parental feeding to the child’s “genetically influenced behavioral profile” [34,79,80,81]. However, little research has sought to examine the relationship between global feeding styles among parents and children’s eating behaviors. Most studies target specific goal-directed feeding practices such as restriction and pressure to eat [5,54,61,62,63,79,82,83,84,85,86]. To our knowledge, this study provides the first data regarding how individual differences in children’s food motivation in the preschool years may interact with mothers’ global feeding styles in predicting children’s subsequent weight status.

Only one interaction was found to be significant, and it was contrary to the hypothesis. Authoritative feeding positively predicted subsequent child weight status but only for children *low* on food motivation. Based on the feeding literature, we expected that feeding styles would be an important predictor for children who were highly motived eaters, given their greater susceptibility to obesity [12,20,28,37,87,88]. Moreover, the finding, that authoritative feeding showed a positive association with subsequent child weight status, was contrary to the literature that mostly supports the premise that authoritative feeding is associated with healthier child weight and eating outcomes [4,79]. This interaction showed that eating motivation moderated the effects of feeding styles on child outcomes, but also suggests that the relationship may be complex and influenced by other factors. Previous research has found that high levels of control in Hispanic parents may play a protective role against negative child health outcomes [10,89]. Some researchers have proposed that controlling interactions in Hispanic families provide the maternal involvement, care, structure, and guidance that children need to develop later autonomy which, in turn, may facilitate positive health outcomes [10,89,90,91,92,93,94]. This suggests that goal-directed feeding practices may play a greater role in these interactions between feeding styles and eating behaviors in this population. Future research should examine how children’s food motivation, maternal feeding styles, and goal-directed feeding practices may interact to contribute to children’s later obesity risk.

Although not the primary focus of this study, monitoring also predicted later child weight. Monitoring during feeding has shown mixed results with child eating [8] and few studies have shown associations with child weight [79,95,96,97]. Similar to Faith and colleagues [98], monitoring in the present study negatively predicted subsequent child weight. Previous studies have also found that parental monitoring has been negatively associated with children eating large amounts of food [99]. However, monitoring was not significantly associated with children’s food motivation in the present study.

Several limitations should be considered in the interpretation of these findings. The study sample was comprised of one ethnic group that encompassed Hispanic families from multiple countries and was recruited from Head Start centers in a large urban US city, thus generalizability is limited. Maternal feeding styles were measured using questionnaires, which can be confounded by social desirability that may have biased mothers’ responses based on expectations rather than actual behavior [100]. The study also has several strengths. Data were collected longitudinally during a critical developmental time for approximately 42 months, starting in preschool, which allowed examination of the target behaviors overtime. Anthropometrics were objectively measured and child behaviors were assessed using both maternal report and observations. Furthermore, all questionnaires were well known and widely used in the feeding literature [7,13,40,60]. Furthermore, the feeding styles questionnaire has been validated by home observations [13,101].

In conclusion, this study showed that food motivation in children interacts differentially with parental feeding and this interaction predicted later child weight status in Hispanic families with low incomes. The current findings highlight the importance for research efforts to progress beyond a one-size-fits all approach to parental feeding. This approach is especially needed to address equity in obesity prevention efforts for families with low incomes who are underrepresented in the feeding literature and for whom prevention efforts have shown limited success [79,102,103,104]. Examining parental feeding by both global feeding styles and goal-directed feeding behaviors within those styles, as well as child eating phenotypes may assist in tailoring childhood obesity prevention programs for maximum benefits for the target population. Researchers have identified the need for characterizing and validating the child eating phenotype in order to better understand the parent/child feeding dynamic and its health outcomes [105]. Future research that targets goal-directed feeding practices among child eating phenotypes may shed light on the results from the current study. Additional research is needed to replicate these findings with larger samples that are ethnically diverse, as well as to evaluate the effectiveness of tailoring programs to children’s unique “profiles”. Furthermore, since within group differences may exist among the Hispanic population, future research should investigate subcultural differences in parental feeding styles.

## Figures and Tables

**Table 1:** Baseline characteristics of the sample.

	Participants (*n* = 129)
Parent gender-female	100.0%
Child gender-female	53.5%
Parent age in years *M* (*SD*)	31.55 (6.60)
Child age in years *M* (*SD*)	4.76 (0.46)
Education of parent	
Less than high school diploma	38.0%
High school diploma or equivalent	24.0%
Some college or more	38.0%
Employment status, currently employed	20.9%
Marital status	
Married	58.9%
Never Married	14.0%
Widowed, separated, divorced	27.1%
Parent immigrant status	
Born in U.S.	17.8%
Born in Mexico	63.5%
Born in Central America	17.9%
Other	0.8%
Child immigrant status	
Born in U.S.	96.9%
Child BMI categories	
Underweight (<5th percentile)	1.6
Healthy (5th to <85th percentile)	48.8
Overweight (85th to <95th percentile)	22.5
Obese (≥95th percentile)	27.1

**Table 2:** Child Eating Behavior Questionnaire (CEBQ) subscale loadings for the highly motivated eating component (single component solution).

Subscale	Loadings
Enjoyment of Food	0.79 *
Emotional Overeating	0.26
Satiety Responsiveness	−0.79 *
Slowness in Eating	−0.54 *
Desire to Drink	0.25
Food Fussiness	−0.62 *
Emotional Undereating	−0.26
Food Responsiveness	0.60 *

* Subscales used to calculate the highly motivated eating score.

**Table 3:** Means, standard deviations, and correlations between study measures (Time 1: *n* = 187 and Time 3: *n* = 129).

	Mean	SD	T1 CFQ Restriction	T1 CFQ Pressure to Eat	T1 CFQ Monitoring	T1 CEBQ Highly Motivated Eating	T1 EAH	T1 CFSQ Authoritative	T1 CFSQ Authoritarian	T1 CFSQ Indulgent	T1 Child BMI *z*-Score	T3 Child BMI *z*-Score
T1 CFQ Restriction ^a^	3.60	0.73	-									
T1 CFQ Pressure to Eat ^a^	3.60	0.87	0.24 ***	-								
T1 CFQ Monitoring ^a^	4.25	0.85	0.12	0.04	-							
T1 CEBQ Highly Motivated Eating ^a^	3.09	0.50	−0.01	−0.08	−0.03	-						
T1 EAH ^b^	2.20	0.75	00.03	−0.13	−0.04	00.04	-					
T1 CFSQ Authoritative ^c^	0.16	0.37	−0.01	−0.05	0.08	0.08	0.02	-				
T1 CFSQ Authoritarian ^c^	0.35	0.48	0.27 ***	0.26 ***	−0.10	−0.32 ***	0.00	−0.32 ***	-			
T1 CFSQ Indulgent ^c^	0.33	0.47	−0.33 ***	−0.21**	0.07	0.24 ***	−00.01	−0.31 ***	−0.52 ***	-		
T1 Child BMI *z*-score	0.94	1.14	−0.03	−0.23 ***	−0.07	0.23 **	0.21 **	0.03	−0.15 *	0.15 *	-	
T3 Child BMI *z*-score	0.94	1.08	0.01	−0.10	−0.22 *	0.16	0.23 **	0.02	−0.14	0.20 *	0.83 ***	-

^a^ 5-point scale; ^b^ 3-point scale; ^c^ 1 = yes, 0 = no; * *p* < 0.05; ** *p* < 0.01; *** *p* < 0.001; T1 = Time 1; T3 = Time 3; CFQ = Child Feeding Questionnaire; CEBQ = Child Eating Behavior Questionnaire; EAH = Eating in the Absence of Hunger; CFSQ = Caregiver’s Feeding Styles Questionnaire; BMI = Body Mass Index.

**Table 4:** Regression analyses of Time 1 maternal feeding style and highly motivated eating in children predicting child BMI *z*-scores at Time 3 controlling for Time 1 child BMI *z*-scores and maternal feeding practices. Columns 2 and 3 present the post-hoc regressions for above and below median scores on highly motivated eating.

	All Participants (*n* = 129)		Below Median Scores on Highly Motivated Eating (CEBQ) (*n* = 64)		Above Median Scores on Highly Motivated Eating (CEBQ) (*n* = 65)	
Model Adjusted *R*^2^ *F* (Model)	0.72 *F*(11,117) = 31.04	***	0.69 *F*(5,58) = 29.02	***	0.68 *F*(5,59) = 27.54	***
Independent Variables	B (95% CI)		B (95% CI)		B (95% CI)	
T1 Child BMI *z*-score	**0.78** (0.69–0.88)	***	**0.83** (0.68–0.98)	***	**0.74** (0.61–0.88)	***
T1 CFQ Restriction ^a^	0.15 (−0.02–0.32)		0.08 (−0.16–0.33)		−0.02 (−0.23–0.19)	
T1 CFQ Pressure to Eat ^a^	0.02 (−0.11–0.15)		−0.03 (−0.26–0.21)		0.04 (−0.14–0.21)	
T1 CFQ Monitoring ^a^	**−0.17** (−0.30–−0.04)	**	**−0.23** (−0.45–0.00)	+	−0.09 (−0.26–0.08)	
T1 CFSQ Authoritative ^b^	**0.39** (0.03–0.75)	*	**0.47** (0.02–0.92)	*	0.02 (−0.34–0.39)	
T1 CFSQ Authoritarian ^b^	−0.10 (−0.43–0.22)					
T1 CFSQ Indulgent ^b^	**0.40** (0.06–0.73)	*				
T1 CEBQ Highly Motivated Eating ^a^	0.34 (−0.26–0.94)					
T1 Authoritative Feeding X Highly Motivated Eating	**−0.96** (−1.77–−0.15)	*				
T1 Authoritarian Feeding X Highly Motivated Eating	−0.43 (−1.13–0.27)					
T1 Indulgent Feeding X Highly Motivated Eating	−0.39 (−1.15–0.36)					

^a^ 5-point scale; **^b^** 1 = yes, 0 = no; + *p* = 0.05; * *p* < 0.05; ** *p* < 0.01; *** *p* < 0.001; Significant results are indicated in bold text; T1 = Time 1; CEBQ = Child Eating Behavior Questionnaire; CFQ = Child Feeding Questionnaire; CFSQ = Caregiver’s Feeding Styles Questionnaire; BMI = Body Mass Index.

## Data Availability

The datasets generated for this article can be requested from the corresponding author, S.O.H.
